# Factors Associated with Violent Behavior among Adolescents in Northeastern Brazil

**DOI:** 10.1155/2014/863918

**Published:** 2014-12-08

**Authors:** Roberto Jerônimo dos Santos Silva, Nara Michelle Moura Soares, Antônio César Cabral de Oliveira

**Affiliations:** ^1^Department of Physical Education, Federal University of Sergipe, 49100-000 São Cristóvão, SE, Brazil; ^2^Department of Health Sciences, Federal University of Sergipe, 49100-000 São Cristóvão, SE, Brazil

## Abstract

The aim of this study was to identify prevalence and factors associated with violent behavior among adolescents in Aracaju and Metropolitan region. The study included 2207 adolescents (16.03 ± 1.08 years old) enrolled in high schools of the State Public Network. Violent behavior was identified from question 14 of the YRBS-2007 questionnaire with responses categorized as “never” and “one or more times.” Higher prevalence in males in relation to risk factors for adoption of violent behavior was found: cigarette consumption (7.3%), alcohol consumption (39.1%), and marijuana use (3.4%). Data analysis used descriptive statistics and logistic regression with hierarchical model at two levels: (a) sociodemographic variables and (b) behavioral variables. For both sexes, association between violent behavior and cigarette smoking (OR = 3.77, CI 95% = 2.06–6.92 and OR = 1.99, CI 95% = 1.04 to 3.81, male and female, resp.) and alcohol consumption (OR = 3.38, CI 95% = 2.22 to 5.16 and OR = 1.83, CI 95% = 1.28 to 2.63, male and female, resp.) was verified. It was concluded that violent behavior is associated with the consumption of alcoholic beverages and cigarettes among adolescents.

## 1. Introduction

The World Health Organization defines violence as the use of physical force or real threat power against oneself, against another person, or against a group or community, which either results in or has any chance of resulting in injury, death, psychological harm, development disability, or deprivation [1, 2]; thus violent behavior can be considered as the behavior that leads the subject or community to commit violent acts.

Thus, violent behavior, especially the use of physical violence, is a significant public health problem worldwide because it has serious personal and social consequences. A greater concern is the group of children and adolescents, they appear in the statistics as the most seriously injured and more murders of occurrences, as suggested by several studies in an attempt to expand the knowledge about the possible factors associated with physical fights among youngsters [1–4].

Previous research has shown a high prevalence of violent behavior through physical struggle. The Health Behavior in School-Aged Children (HBSC) [4] identified prevalence of physical fights in 14% of young people aged 13 and older in the European Union. In the United States, the Youth Risk Behavior Survey (YRBS) identified that 34.4% of high school students (approximately 16 years old) have been involved in some sort of physical fight [5].

In Brazil, a study carried out in the southern region showed that 16.5% of young people (15 to 19 years old) have been involved in physical fights in the 12 months preceding the survey [6]. The National Survey on Students' Health (PeNSE) [7] conducted with adolescents (13 to 15 years old) of the ninth grade of Brazilian state capitals and the Federal District showed a prevalence of 12.9% of reported physical fight in the 30 days preceding the survey. However, the present study is to aggregate information in order to show that, in Brazil, the violence behavior may follow the same trend with high prevalence.

The inappropriate use of alcohol and other drugs may be related to violent behavior, but to assess this relationship we must consider multiple variables during assessments. Thus, it appears that although there is a population-based survey [7] in Brazil warning of these behaviors, there is still little information regarding violent behavior, especially in regard to physical fights among adolescents and associated factors. It is noteworthy that this information is presented as extremely important by favoring actions in terms of public policies aimed at this population.

Studies in which adolescents are the focus of observation favor objective interventions for this group from the appropriate mapping considering the area and location of the observation and clear actions on each risk factor, thus reducing the prevalence over the school years of these young people.

Based on the above, this study aims to identify the prevalence and factors associated with violent behavior among high school students of Aracaju and Metropolitan region.

## 2. Methods

### 2.1. Study Design and Sample Size Calculation

This sectional descriptive school-based study included students enrolled in high schools of Aracaju and Metropolitan Region that make up 40.42% of all high school students in the State of Sergipe, Brazil.

The school population base was composed of 13,373 students enrolled in the year 2011 in high schools of the State Public Network of districts of Aracaju, Nossa Senhora do Socorro, Barra dos Coqueiros, and São Cristovão.

The sample was defined using the cluster sampling in two-stage procedure, considering the administrative distribution according to Regional Departments of Education used by the State Department of Education of the State of Sergipe. In the first stage, high schools of each municipality were considered conglomerates. The number of enrollments should be higher than 350 students, totaling 19 schools across the region observed, with a total of 13373 students.

The second stage was characterized by sample stratification by Teaching Unit, considering the classroom as the sampling unit. The objective was to achieve the minimum number of students required for the performance of the study as well as the amount of classrooms that would be drawn considering the criteria of proportionality by grade.

The sample size calculation followed the suggested procedures for cluster type sampling by adding 20% to the calculated amount of subjects in order to compensate losses and refusals [8]. Thus, the minimum number of subjects to compose the sample, considering a sampling error of 3.0 percentage points and an expected prevalence of 14% [6] of observed behaviors with effect design of 2.0, was 1195 subjects.

### 2.2. Inclusion Criteria and Data Collection

The entire survey occurred in three months, having started in July 2011. Inclusion criteria were as follows: (a) being regularly enrolled in high school of Teaching Units chosen to participate in the study; (b) having minimum age of 13 and maximum of 18 years; (c) having delivered the informed consent form (ICF) signed by parent or guardian; (d) being present on the day of data collection.

Each Teaching Unit selected was visited two times. On the first day, the objectives of the study were explained to clarify doubts and to deliver the ICF to students so that parents or guardians could sign it authorizing their participation. Data collection took place on the second day.

The staff was composed of 15 researchers (ten physical education students of the Federal University of Sergipe and five health professionals). All researchers were trained for a month, where standardization and proper use of the instrument used were detected. After training, a pilot study was done.

The instrument used was compiled from three other self-report instruments already validated in Brazil. For the definition of economic class and education level of parents or guardians, the questionnaire developed by ABEP [9] was used. The classification of socioeconomic level was grouped into the following strata: “high” (“A1,” “A2,” “B1,” and “B2”), “intermediate” (“C1” and “C2”), and “low” (“D” and “E”).

Maternal and paternal educational level was stratified into “illiterate to 3rd grade,” “incomplete basic education,” “complete basic education,” “complete high school,” and “complete higher education,” as suggested in the methodology used by ABEP [10].

To characterize the outcome “violent behavior” and risk behaviors (dependent variables) “cigarette smoking,” “alcohol consumption,” and “marijuana consumption,” questions “18,” “30,” “41,” and “47” of the YRBS-Brazil questionnaire were used [9].

Violent behavior was verified by question “18”: “During the past 12 months, how often did you get involved in a physical fight?” For consumption of tobacco, alcohol, and marijuana, questions “30” (During the past 30 days, on how many days did you smoke cigarettes?), “41” (During the past 30 days, on how many days you took at least one dose alcoholic drink?), and “47” (During the past 30 days, on how many days did you use marijuana?) were used. The responses were categorized into two groups, “never” and “one or more times,” respectively called “no” and “yes” in this survey.

As directed by the IBGE [7], the weekly time the adolescent spent watching TV was also asked and quantified with the following question: “On average, how many hours per day do you spend watching television?”

### 2.3. Data Analysis

Descriptive statistics was used to characterize the study group. Multivariable logistic regression was conducted in order to verify associations in crude and adjusted analyses, as needed, estimating odds ratios and confidence intervals of 95%.

The hierarchical model was built on two levels: the first level included sociodemographic variables (gender, age, socioeconomic level, maternal and paternal education, and ethnicity) and the second level included variables that characterize risk behaviors (cigarette smoking, alcohol consumption, and marijuana consumption). The possibility of confounding and interaction was considered in the study from specific analysis. Significance level of 5% (*P* < 0.05) was used in all analyses. All procedures were performed using the SPSS software version 19.0 for Windows.

## 3. Results

Overall, 2457 questionnaires were applied: (a) 133 (5.41%) were completed by individuals aged 18 years or older; (b) 8 (0.33%) were completely blank; (c) 30 (1.22%) were missing important data such as gender and age; (d) 22 (0.9%) had less than 2/3 of answered questions; and 57 (2.32%) did not have parental permission to complete the instrument, totaling 250 (10.18%) lost or discarded questionnaires. Thus, 2207 (89.82%) questionnaires were eligible for this data collection, of which 836 (37.9%) were of males.

Table 1 presents the characteristics of the sample in relation to socioeconomic variables and risk behaviors. The high prevalence of alcohol consumption (39.1%) and involvement in physical fights of one or more times in the last 12 months (26.3%) in male attracts attention. It was found that violent behavior is more prevalent among boys, affecting about one-fourth of the subjects.

Table 2 shows crude and adjusted associations between variables observed in the study for females. It should be reported that no associations between socioeconomic variables and violent behavior were observed; however, it was found that risk behaviors, cigarette smoking (OR = 6.83, CI 95% 3.96 to 11.77), alcohol use (OR = 4.19, CI 95% 2.78 to 6.30), and marijuana use (OR = 7.00, CI 95% 2.89 to 17.00), were associated with violent behavior in the crude model, observing that adolescents who consumed cigarettes were almost seven times more likely to exhibit this behavior in relation to those who did not consume alcohol, with a similar result for those who used marijuana. Adolescents who consume alcohol were four times more likely to show violent behavior compared to the reference group.

When associations adjusted for females between violent behavior and other dependent variables were observed (Table 2), it appears that there is an association only with cigarette smoking (OR = 3.77, CI 95% = 2.06–6.92) and alcohol consumption (OR = 3.38, CI 95% 2.22 to 5.16). Interestingly, in both variables, the adoption of these behaviors together increases by just over three times the chance of adopting violent behavior.

In relation to males (Table 3), no correlations between violent behavior and socioeconomic variables were found, in which the crude model showed association between violent behavior and cigarette smoking (OR = 3.32; CI 95% = 1.85 to 6.00), alcohol consumption (OR = 2.16, CI 95% 1.53 to 3.04), and marijuana consumption (OR = 4.05, CI 95% = 1, 70 to 9.64), indicating how these behaviors can change the behavior of male adolescents.

For the adjusted model, the same trend of association with female gender was observed; that is, the behavior of cigarette consumption (OR = 1.99, CI 95% 1.04 to 3.81) and alcohol consumption (OR = 1.83, CI 95% 1.28 to 2.63) is associated with violent behavior, increasing at around twice the chance of adolescents to show these behaviors (Table 3).

## 4. Discussion

This study aimed to identify prevalence and factors associated with violent behavior among adolescents in Aracaju, northeastern Brazil, and the implication of this study is to subsidize interventions to minimize the prevalence of this behavior.

### 4.1. High Prevalence of Violent Behavior for Males

Thus, higher prevalence of violent behavior among males was observed, as well as association between this behavior and cigarette smoking and alcohol consumption, regardless of sex. This result alone indicates that these variables are associated with violent behavior and need to be the focus of the intervention.

It could also be observed that 26.3% of boys and 10.4% of girls have been involved in physical fights. This suggests a greater propensity of male adolescents to try to meet their needs getting involved in fights. Regarding risk behaviors by gender, almost the same prevalence of cigarette smoking and alcohol consumption was observed, and boys have twice the reported consumption of marijuana of the girls.

The results concerning the prevalence of physical fighting among youngsters in Sergipe agree with those observed in a study intended to estimate the prevalence of physical fighting and associated factors among 13857 American adolescents, where prevalence of 13.5% of young people involved in physical fights was detected, with higher prevalence among boys (18.2%) [2]. These findings suggest that boys are the ones who use violence to resolve interpersonal conflicts.

A cross-sectional study with 15,448 adolescents held in Hong Kong using the same instrument of this study for data collection found lower prevalence of violent behavior among groups in which parents were better educated, suggesting influence of the level of parental education on violent behavior among adolescents [11]. However, in the present study, no association between parental education and violent behavior of students was identified.

In a population-based study conducted in the United States aimed at analyzing trends and determining the progress towards the goals of Healthy People 2010 using the same questionnaire used here considering the 1999–2009 period, it was observed that there was a reduction from 35.7 to 31.5 in the prevalence of physical fights over the observation period [12], with higher prevalence compared to youngsters from Sergipe. Interestingly, there are no studies in Brazil that have followed this trend of longitudinal observation, which represents a public health need to characterize the possibilities for change in behavior with mistakes and successes, indicating paths to be followed by public policies aimed at these youngsters.

A study in Portugal [13] showed that involvement in physical fights is related to alcohol consumption, smoking, and age at first intercourse, agreeing with this study by stating that the consumption of alcohol and smoking are risk factors for the adoption of violent behavior.

The data reported here are similar to those observed in a study on factors associated with violent behavior in 699 students of public schools in Midwestern Brazil, which found a prevalence of 20.2% of violent behavior among adolescents aged 12–19 years and a strong association between this behavior and males, alcohol consumption, use of psychoactive drugs, and poor relationships with parents [14].

### 4.2. Risk Factors for Violent Behavior

Association between violent behavior and cigarette smoking in both crude and adjusted model and in both sexes was found, and girls who reported cigarette consumption were almost twice more likely to get involved in fights than boys.

Considering the adjusted model, Tables 2 and 3 show that, in regard to alcohol consumption, girls are almost twice more likely to get involved in fights than boys. An interesting fact to be observed is that females are more likely to have violent behavior for the same variables, suggesting greater attention.

This association follows the same trend as the association between fights/violent behavior and alcohol consumption, in which adolescents who consume alcohol are 1.5 times more likely to get involved in fights [2]. This explains why the individual who consumes alcohol is more likely to commit acts of violence and also makes him/her more vulnerable to violent conduct of other persons. Although the associations found between alcohol consumption and cigarette smoking with violent behavior give rise to speculations, Garbarino [15] tries to explain the causes of violent behavior among young people from an ecological perspective, arguing that, necessarily, there is no cause and effect relationship to explain violent behavior among adolescents.

From this perspective, before conclusions to explain/justify violent behavior, it is necessary to consider variables such as gender, temper, cognitive ability, age, family, neighborhood, country/society, and culture in which the subject is involved; that is, it is not a matter of positive or negative influence, but rather a set of factors that lead young people to have violent behavior [15].

Some authors have discussed the influence of media on these individuals, emphasizing that the references made by the media about actions related to violence where characters linked to violence are presented almost like celebrities for audience tend to reverse values related to violence in some groups [16]. 

*Limitations.* The use of questionnaires that, due to limitations arising from some questions, even guaranteeing anonymity, can hamper the accuracy of some information in some subjects can be pointed out as a limitation of this study.

Another limitation is the extrapolation of these results only to schooled young people, attending to the need for a survey including unschooled young people as a way to subsidize actions aimed at this group.

Thus, considering the objectives proposed by the study, it was concluded that, in adolescents enrolled in public schools of the study region, violent behavior is associated with alcohol and cigarette consumption.

Interventions in the school environment should be implemented, either in the continued preparation of teachers, in the development of curricula where these issues comprise the day-to-day lives of students, or in the awareness of adolescents in order to minimize the prevalence of these behaviors.

## Figures and Tables

**Table 1:** Socio-demographic characteristics and prevalence of violent behavior/physical fights among adolescents, State of Sergipe, Brazil, *n* = 2207.

Variable	Whole *n* (%)	Female *n* (%)	Male *n* (%)
Gender	—	1371 (62.1)	836 (37.9)

Age (years)			
14	99 (4.5)	55 (4.0)	44 (5.3)
15	476 (21.6)	302 (22.0)	174 (20.9)
16	684 (31.1)	429 (31.4)	255 (30.6)
17	638 (29.0)	403 (29.5)	235 (28.2)
18	302 (13.7)	177 (13.0)	125 (15.0)

Income level			
High	517 (23.7)	283 (20.9)	234 (28.4)
Medium	1383 (63.4)	879 (64.8)	504 (61.2)
Low	280 (12.8)	194 (14.3)	86 (10.4)

Maternal education			
Illiterate to 3rd grade	141 (6.7)	96 (7.3)	45 (5.7)
Incomplete basic education	589 (27.9)	378 (28.8)	211 (26.6)
Complete basic education	569 (27.0)	365 (27.8)	204 (25.7)
Complete high school	679 (32.2)	398 (30.3)	281 (35.4)
Complete higher education	130 (6.2)	77 (5.9)	53 (6.7)

Paternal education			
Illiterate to 3rd grade	161 (8.0)	122 (9.7)	39 (5.2)
Incomplete basic education	567 (28.3)	379 (30.2)	188 (25.0)
Complete basic education	501 (25.0)	288 (23.0)	213 (28.3)
Complete high school	656 (32.7)	399 (31.8)	257 (34.1)
Complete higher education	121 (6.0)	65 (5.2)	56 (7.4)

Cigarette consumption in past 30 days			
No	2051 (93.4)	1277 (93.8)	774 (92.7)
Yes	146 (6.6)	85 (6.2)	61 (7.3)

Alcohol drinking in past 30 days			
No	1350 (61.5)	845 (61.9)	505 (60.9)
Yes	844 (38.5)	520 (38.1)	324 (39.1)

Marijuana consumption in past 30 days			
No	2140 (97.6)	1339 (98.2)	801 (96.6)
Yes	53 (2.4)	25 (1.8)	28 (3.4)

Hours watching TV			
Up to 2 hours	648 (30.9)	385 (29.1)	263 (33.8)
More than two hours	1451 (69.1)	936 (70.9)	515 (66.2)

Violent behavior: Involvement in physical fights			
Never	1842 (83.5)	1227 (89.6)	615 (73.7)
One or more times	363 (16.5)	143 (10.4)	220 (26.3)

**Table 2:** Association between violent behavior and associated factors among female adolescents. State of Sergipe, Brazil.

Variable	Crude OR (CI 95%)	*P*	Adjusted OR^*^ (CI 95%)	*P*
Age (years)				
14	1.15 (0.31–4.29)	0.58	—	—
15	0.70 (0.33–1.45)			
16	0.55 (0.27–1.08)			
17	0.69 (0.34–1.39)			
18	1			

Income level				
High	0.60 (0.30–1.17)	0.10		—
Medium	0.81 (0.44–1.49)		—	
Low	1			

Maternal education				
Illiterate to 3rd grade	1.37 (0.54–3.51)	0.09		—
Incomplete basic education	1.97 (0.91–4.29)			
Complete basic education	1.71 (0.79–3.69)		—	
Complete high school	1.21 (0.58–2.54)			
Complete higher education	1			

Paternal education				
Illiterate to 3rd grade	1.64 (0.64–4.21)	0.10	—	—
Incomplete basic education	1.90 (0.85–4.24)			
Complete basic education	1.91 (0.83–4.37)			
Complete high school	1.29 (0.59–2.79)			
Complete higher education	1			

Cigarette consumption in past 30 days				
No	6.83 (3.96–11.77)	<0.001	3.77 (2.06–6.92)	<0.001
Yes	1		1	

Alcohol drinking in past 30 days				
No	4.19 (2.78–6.30)	<0.001	3.38 (2.22–5.16)	<0.001
Yes	1		1	

Marijuana consumption in past 30 days				
No	7.00 (2.89–17.00)	<0.001	1.73 (0.65–4.62)	0.28
Yes	1		1	

Hours watching TV				
Up to 2 hours	1.08 (0.71–1.64)	0.73	—	—
More than two hours	1			

^*^Model adjusted for “cigarette consumption in past 30 days”, “alcohol consumption in past 30 days”, “marijuana consumption in past 30 days.”

**Table 3:** Association between violent behavior and associated factors among adolescent males. State of Sergipe, Brazil.

Variable	Crude OR (CI 95%)	*P*	Adjusted OR^*^ (CI 95%)	*P*
Age (years)				
14	0.58 (0.25–1.32)	0.12	—	—
15	0.64 (0.36–1.14)			
16	0.70 (0.41–1.21)			
17	0.73 (0.42–1.27)			
18	1			

Income level				
High	0.63 (0.33–1.19)	0.14		—
Medium	0.74 (0.40–1.37)		—	
Low	1			

Maternal education				
Illiterate to 3rd grade	0.96 (0.38–2.41)	0.95		—
Incomplete basic education	1.12 (0.55–2.30)			
Complete basic education	0.85 (0.42–1.73)		—	
Complete high school	1.06 (0.52–2.12)			
Complete higher education	1			

Paternal education				
Illiterate to 3rd grade	1.65 (0.64–4.27)	0.18	—	—
Incomplete basic education	1.82 (0.94–3.55)			
Complete basic education	1.62 (0.84–3.11)			
Complete high school	1.55 (0.82–2.92)			
Complete higher education	1			

Cigarette consumption in past 30 days				
No	3.32 (1.85–6.00)	<0.001	1.99 (1.04–3.81)	0.04
Yes	1		1	

Alcohol drinking in past 30 days				
No	2.16 (1.53–3.04)	<0.001	1.83 (1.28–2.63)	<0.01
Yes	1		1	

Marijuana consumption in past 30 days				
No	4.05 (1.70–9.64)	<0.01	2.11 (0.84–5.33)	0.11
Yes	1		1	

Hours watching TV				
Up to 2 hours	1.31 (0.91–1.88)	0.15	—	—
More than two hours	1			

^*^Model adjusted for “cigarette consumption in past 30 days”, “alcohol consumption in past 30 days”, “marijuana consumption in past 30 days.”
